# Observation and manipulation of glial cell function by virtue of sufficient probe expression

**DOI:** 10.3389/fncel.2015.00176

**Published:** 2015-05-08

**Authors:** Akiyo Natsubori, Norio Takata, Kenji F. Tanaka

**Affiliations:** Department of Neuropsychiatry, School of Medicine, Keio UniversityTokyo, Japan

**Keywords:** knockin-mediated enhanced gene expression, tet system, tTA, tetO, optogenetic actuator, calcium indicator, genetically modified mice

## Abstract

The development of gene-encoded indicators and actuators to observe and manipulate cellular functions is being advanced and investigated. Expressing these probe molecules in glial cells is expected to enable observation and manipulation of glial cell activity, leading to elucidate the behaviors and causal roles of glial cells. The first step toward understanding glial cell functions is to express the probes in sufficient amounts, and the Knockin-mediated ENhanced Gene Expression (KENGE)-tet system provides a strategy for achieving this. In the present article, three examples of KENGE-tet system application are reviewed: depolarization of oligodendrocytes, intracellular acidification of astrocytes, and observation of intracellular calcium levels in the fine processes of astrocytes.

## Expressing Functional Genes to Observe and Manipulate Glial Cells

To observe and manipulate glial cell functions, probes needed for selective observation and manipulation of cells should be expressed in advance. These functional probes include the intracellular calcium sensor molecules such as GCaMP (Nakai et al., 2001) for observation, and light-sensitive membrane potential converting molecules, such as channelrhodopsin for depolarization (Nagel et al., 2003) and halorhodopsin (HaloR; Zhang et al., 2007) or archaerhodopsin (Arch)/ArchT (Chow et al., 2010; Han et al., 2011) for hyperpolarization. Mouse Genetics can be used for glial cell-specific expression of these molecules.

Glial cells are present in every brain region and show complex interactions with neurons. Therefore, when observing or manipulating glial cell functions, glial cell-specific expression of functional molecules is vital. These functional molecules should be fully expressed in transfected glial cells in a cell type-specific manner to exert their activities. In general, however, it is not easy to simultaneously achieve both a high level and a cell type-specific expression. The worst-case scenario would be a failure to express these molecules at a level sufficient to exert their functions, as a result of too much focus placed on the cell type specificity. For example, channelrhodopsin 2 (ChR2) molecules were successfully expressed specifically in astrocytes, but failed to trigger photocurrent because of low expression. To continue with glial cell research, cell type-specific expression of functional probes need to be pursued, while simultaneously avoiding such undesirable scenarios.

To accomplish cell type-specific expression of functional probes in the brain, many researchers have employed local viral injections as a means to introduce those genes. However, viral injection via a needle causes cerebral parenchyma injury and will induce substantial alterations in the nature of glial cells. Injury-induced augmentation of glial cell activity and subsequent cross-interactions between glia and neurons are inevitable to some degree. Therefore, any research projects that cannot disregard the effects of external injury need alternative methods. If mouse genetics were adapted to this purpose, functional molecules could be expressed without any problems caused by external injuries.

## Making History of KENGE-tet

What are the possibilities for reasonable expression of functional probes specifically in glial cells using mouse genetics? We have tried glial cell-specific expression of ChR2 using transgenic mouse technology. We attempted not only to achieve glial cell-specific expression of ChR2, but also to fulfill high enough expression to function, in this case to induce photocurrent.

ChR2 conductance was reported to be 50–250 fS (Lin et al., 2009), indicating a smaller conductance compared to those of most ionic channels reported until then. This fact suggested that a “more than a moderate level of expression” of ChR2 would be needed to control the membrane potential. Although this information had been recognized early, scientists did not know how much ChR2 should be expressed on the cell membrane to successfully control the membrane potential. Higher level of expression was considered to be better than less expression. However, aggregation of ChR2 molecules was a concern for having too much expression. Scientists were confused by the variety of information. Furthermore, it was suggested that ChR2 molecules stayed in the endoplasmic reticulum and did not reach the cell membrane causing failure of detection of photocurrents. To evaluate this possibility, expression of a ChR2-green fluorescent protein (GFP; or mCherry) fusion protein was attempted. This made the already difficult situation more difficult. Expression of a ChR2-GFP (or mCherry) fusion protein was a challenging subject when compared to expression of ChR2 molecules alone.

**Figure 1:**
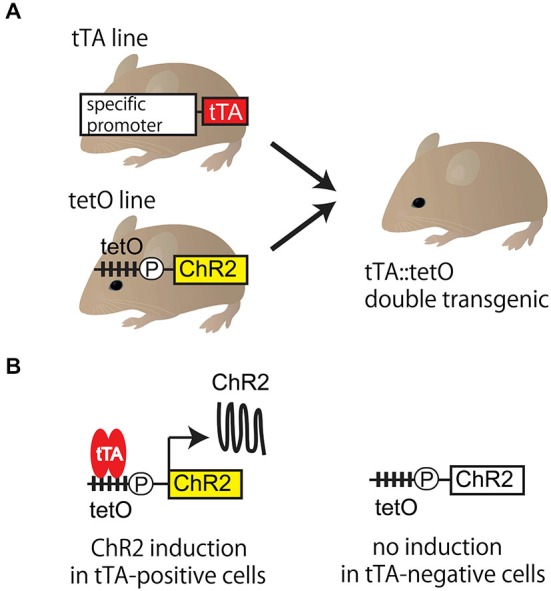
**Tet system. (A)** Tet system requires two mouse lines. The first is a mouse line expressing gene encoding tetracycline transactivator (tTA) protein under the control of cell-type specific promoter. The second is one expressing the functional probe (such as ChR2) connected downstream of the tetO promoter. Targeted tTA::tetO double transgenic mouse is generated by mating. **(B)** The functional probe is expressed in tTA-positive cells, but not in tTA-negative cells in tTA::tetO double transgenic mouse. In tTA-positive cells, tTA binding to the tetO promoter triggers the transcription, which results in the cell-type specific expression of ChR2. TetO is the cassette containing the tetracycline operon site (multiple closed squares) and CMV minimal promoter (circle P). ChR2: channelrhodopsin 2, tetO: tet operator, tet system: tetracycline-controllable gene expression system, tTA: tetracycline transactivator.

The tet system (tetracycline-controllable gene expression system) is a gene expression system (Gossen and Bujard, 1992) allowing enhanced expression of an exogenous gene in a cell type-specific manner. The tet system is a bipartite system (Figure 1). The first mouse line expresses the gene encoding tetracycline transactivator (tTA) protein under the control of cell-type specific promoter. In the second mouse line, ChR2 complementary DNA (cDNA) is connected downstream of the tet operator (tetO) promoter, which is activated only by the presence of tTA protein. Cell-type specific expression of ChR2 can be expected only in tTA::tetO double transgenic mice. If any undesirable events, such as toxicity, are caused by the overexpression, the amount of expression can be lowered using doxycycline, a tetracycline analog.

We first generated tetO-ChR2-mCherry plasmid transgenic lines (actually tetO-ChR2-mCherry, HaloR-GFP lines) and established 6 lines (Chuhma et al., 2011). When we crossed them with neuronal tTA line (*Camk2a*-tTA, Jax stock number 003010), we succeeded in functional ChR2-mCherry expression with all combination, indicating that tet system worked. However, when we crossed them with astrocytic tTA line (*Mlc1*-tTA; Tanaka et al., 2010), Riken BioResource Center (BRC) number 05450), none of combination yielded a red fluorescence in astrocytes. We observed a few HaloR-GFP expressing Bergmann glia in line 6 (Tanaka et al., 2012, Jax stock number 017906), which was the best line among them. The chromosomal positional effect on the tetO transgene by the random insertion, the copy number of tetO transgene, or the DNA methylation of tetO promoter may account for the cell-type dependent difference of gene induction.

**Figure 2:**
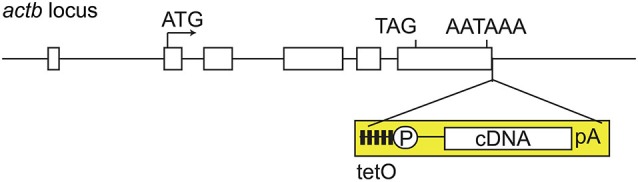
**KENGE-tet system requires tetO cassete knockin**. *Actb* gene structure and insertion site of tetO cassette. Mouse *Actb* gene consists of 6 exons (rectangles), and the tetO cassette (yellow) is inserted downstream of the polyadenylation signal of *Actb* gene. TetO is the cassette containing the tetracycline operon site (closed squares) and CMV minimal promoter (circle P). AATAAA is a polyadenylation signal. Knocking-in of tetO cassette into the euchromatic locus is a key point for the achievement of higher expression with tTA. *Actb*: β-actin, cDNA: complementary DNA, pA: ployadenilation signal, tetO: tet operator, tTA: tetracycline transactivator.

We then attempted to improve the tet system. What was the reason why the tetO promoter failed to induce ChR2 expression despite the fact that expression of tTA was obtained? For this question, we got ideas from our own experience (Tanaka et al., 2010). In past experiments, we established tetO mice by microinjection of transgenic plasmid DNA into the fertilized eggs, resulting in poor tTA-mediated gene induction. However, we found that the efficiency of tTA-mediated gene induction was improved dramatically when tetO mice were generated by homologous recombination technique through embryonic stem cells. From this, we generated the tetO-ChR2 mouse not by transgenic but by knocking-in approach, called the Knockin-mediated ENhanced Gene Expression (KENGE)-tet (Tanaka et al., 2012). The house-keeping β-actin gene locus was used as the knock-in site and the tetO-ChR2 cassette was inserted (Figure 2). This strategy was a major success and tremendously high levels of ChR2 expression was achieved compared to the previous tetO lines. Importantly, the levels of ChR2 in astrocytes, oligodendrocytes, and microglia were high enough to trigger photocurrents with *Mlc1*-tTA, *Plp1*-tTA (Inamura et al., 2012, Riken BRC number 05446), and *Aif1*-tTA (also known as *Iba1-*tTA; Tanaka et al., 2012, Riken BRC number 05769) lines, respectively.

KENGE-tet is not limited in the usage of β-actin gene locus. The usage of euchromatic locus, such as collagen type 1 alpha 1 gene (*Col1a1*) locus (Beard et al., 2006; Egli et al., 2007; Carey et al., 2010; Premsrirut et al., 2011) or TIGRE (tightly regulated) locus (Zeng et al., 2008; Madisen et al., 2015) shares the same idea of KENGE-tet and yields high levels of tTA-mediated gene expression.

## Expression of Functional Probes using Genetically Modified Mice

In addition to the KENGE-tet sysetm, an improved Cre-loxP system can be used to satisfy the requirements of both a high level and cell type-specific expression. The combination of the euchromatic locus with the stronger promoter greatly enhanced the expression levels of functional probes. The Ai (Allen Institute) mouse (Madisen et al., 2010, 2012), with a CAG promoter inserted in the ROSA26 locus, and the PC (*Polr2a*-CAG) mouse (Gee et al., 2014), with a CAG promoter inserted in the polymerase (RNA) II (DNA directed) polypeptide A (*Polr2a*) gene locus, have been developed as loxP lines.

Both the tet and Cre-loxP systems use double-transgenic mice obtained by mating two mouse lines. One mouse line is aimed at ensuring a cell type-specific expression (Cre or tTA mice), and the other to obtain a high expression level (ROSA-CAG/Polra2-CAG or KENGE-tet mice). LoxP or tetO line has been further improved, providing a wider repertoire of functional probes to be expressed.

Table 1 shows the repertoire of mouse lines for the expression of functional molecules in a glial cell-specific manner (tTA mice), and those for the expression of sufficient amounts of functional molecules (KENGE-tet mice). tTA and tetO mice can be mated in any combination. Although not all the theoretically obtainable mouse lines have been tested, we have confirmed that sufficient expression was obtained for photocurrent induction in astrocyte-specifically expressed ChR2 and ArchT, oligodendrocyte-specifically expressed ChR2 and ArchT, and microglia-specifically expressed ChR2. Yellow cameleon nano50 (YC; Horikawa et al., 2010) was also expressed in an astrocyte-specific manner to a level sufficient for the observation of intracellular calcium level *in vivo*. Most of these mouse lines can be obtained from Riken BioResource Center.^1^ Ai mice are available from the Jackson Laboratory.^2^

**Table 1:** **The repertories of glia-specific tTA lines and KENGE-tetO lines**.

Glial tTA lines				
Target cell	Line name	Ref	RIKEN BRC stock number	Crossing with KENGE-tet mouse
Astrocyte	Mlc1-tTA	Tanaka et al. (2010)	05450	Okada et al. (2012), Sasaki et al. (2012), Tanaka et al. (2012), and Beppu et al. (2014)
	Slc1a2-tTA (GLT1-tTA)	Tanaka et al. (2013)	–	–
	Gfap-tTA	Pascual et al. (2005)	–	–
Oligodendrocyte	Plp1-tTA	Inamura et al. (2012)	05446	Inamura et al. (2012), Tanaka et al. (2012), and Yamazaki et al. (2014)
Oligodendrocyte progenitor cell	Sox10-rtTA	Ludwig et al. (2004)	–	no induction with tetO-ChR2 (C128S) (Inamura et al., 2012)
Microglia	Aif1-tTA (Iba1-tTA)	Tanaka et al. (2012)	05769	Tanaka et al. (2012)
**KENGE-tetO lines**				

**Probe**	**Line name**	**Ref**	**RIKEN BRC stock number**

ChR2 (C128S)	tetO-ChR2 (C128S)	Tanaka et al. (2012)	05454
ChR2 (E123T/T159C)	tetO-ChR2 (E123T/T159C)	Tsunematsu et al. (2014)	05843
ArchT	tetO-ArchT	Tsunematsu et al. (2013)	05842
YC nano50	tetO-YC	Kanemaru et al. (2014)	–

*(–): not available.tTA: tetracycline transactivator, KENGE: Knockin-mediated ENhanced Gene Expression, GLT-1: glial glutamate transporter-1, Iba1: Ionized calcium-binding adapter molecule 1, ChR2: channelrhodopsin 2, ArchT: archaerhodopsin from Halorubrum strain TP009, YC: Yellow cameleon nano50*.

## Manipulation and Observation of Glial Cells Avoiding Injury

Using genetically modified mice, functional molecules can be expressed specifically in glial cells without injury. However, it is meaningless if the brain injury occurs in the process of observation and manipulation (Xu et al., 2007). What are the possibilities of carrying out observations and manipulations without any injuries? It was demonstrated that mice expressing ChR2(C128S) in astrocytes responded to blue light illumination over the skull (Tanaka et al., 2012), since ChR2(C128S) variant had higher photosensitivity (Berndt et al., 2009). These data indicated that cortical astrocytes could be manipulated without an insertion of optical fiber, but the optic fiber insertion was still required when astrocytes in the deep brain were targeted.

To visualize the effects of optogenetic manipulation, the laser speckle method can be used to observe blood stream changes through the cranial bone (Zakharov et al., 2009), and functional magnetic resonance imaging (MRI) can be used to observe the blood oxygenation level-dependent (BOLD) signal changes from the intact brain (Ogawa et al., 1990). Intracellular calcium concentrations in glial cells *in vivo* can be observed using two-photon microscopy (Hirase et al., 2004; Wang et al., 2006; Schummers et al., 2008; Takata et al., 2011; Nimmerjahn and Bergles, 2015), in which brain parenchyma injuries can be minimized. The process of combining these manipulation and observation methods does not cause injuries to the brain parenchyma, avoiding unwanted glial cell activation.

Glial cells respond to injuries. If you compare this response to a “scream,” and responses during normal interactions between glia and neurons to a “whisper,” then the most intriguing scientific phenomenon for glia researchers is the “whisper” being washed out by the “scream”. To date, there is no definitive evidence indicating responses to injury are extensive and normal responses are imperceptible. However, glia researchers need to implant functional molecules into glial cells like a “spy” to extract information and to alert them to any tiny response in glial cells. We believe that only through these types of efforts we can understand the glial cell function.

## Functional Analysis of Glial Cells after Optogenetic Manipulation

What are the purposes of ChR2-mediated manipulation in glial cells? The purposes of optogenetic manipulation are clear in the case of neurons. In neurons, an inward current generated by light opens voltage-dependent sodium channels at the axon initial segment and induces an action potential. Therefore, ChR2 expressing neurons can generate action potentials on demand. In glial cells, however, action potentials cannot be generated by depolarization. Apart from action potential generation, expected approaches to glial cells using ChR2 are indicated in the following sections:

### Depolarization of Oligodendrocytes by ChR2

Hippocampal slices are often used in experiments for induction of long-term potentiation (LTP) of synaptic transmission. When high-frequency stimulation was delivered to the slice to induce LTP, the depolarization of oligodendrocytes at about 15–20 mV was observed (Yamazaki et al., 2007). The effects of oligodendrocytic depolarization on neuronal activity were investigated from the viewpoint of the oligodendrocyte-neuron interaction. To directly demonstrate the physiological implication of oligodendrocytic depolarization, Yamazaki et al. depolarized a single oligodendrocyte at the alveus of the hippocampus using a glass electrode (Yamazaki et al., 2007). The response was recorded from a single CA1 pyramidal neuron around which clamped-oligodendrocyte wrapped. An action potential was generated by electrostimulation of the distal axon, which traveled retrogradely, and was recorded in the soma of CA1 neuron. The clamped-oligodendrocyte was located between the sites of stimulus and observation. The latency between electrostimulation of the distal axon and detection of the action potential reflects the axonal conduction velocity. Normally the latency, or conduction velocity, is constant, but oligodendrocytic depolarization decreased the latency and increased the conduction velocity of action potentials. These findings suggested that oligodendrocytes regulated the neuronal conduction velocity through the depolarization of oligodendrocytes themselves.

Multiple oligodendrocytes wrap the single axon, therefore, the single axonal activity should affect multiple oligodendrocytes. However, it is technically difficult to mimic such situation by using multiple glass electrodes. To achieve this, Yamazaki et al. constructed an experimental system for simultaneous multiple oligodendrocytic depolarizations by optogenetical approach (Yamazaki et al., 2014). That system using *Plp1*-tTA::tetO-ChR2(C128S) double transgenic animals enabled −20 mV oligodencrocytic depolarization without high-frequency electrical stimulation. They investigated if multi-cellular optogenetic manipulation could induce an increase in the conduction velocity similar to single-cellular glass electrode-mediated depolarization. The results showed a transient (~10 min) but increased conduction velocity. The disappearance of this effect coincided with the termination of oligodendrocytic depolarization.

Yamazaki et al. (2007) also investigated axon excitability using the extracellular recordings. In these recordings, axon excitability was measured as a compound action potential (CAP). Immediately after the illumination of oligodendrocytes, CAPs significantly increased and this effect continued for 3 h. These results suggested either an increased number of excited axons, or increased action potentials from each axon. Interestingly, transient oligodendrocytic depolarization resulted in the plastic change of the nerve conduction for several hours. The oligodendrocytic depolarization by an optogenetic approach recapitulated the response to high-frequent neuronal firing and these data demonstrated that the oligodendrocytic reaction resulted in short- and long-term plasticity of axons.

### Astrocytic Intracellular pH Control by Opsins

Since astrocytes have small membrane resistance, their membrane potential changes very little, even when a photocurrent is delivered. Despite this fact, it was demonstrated that photostimulation of ChR2 expressing astrocytes resulted in the excitation of adjacent neurons (Okada et al., 2012). Using ChR2 expressing Bergmann glial cells of the cerebellum, Sasaki et al. revealed that (1) glutamate was released from Bergmann glia during ChR2 photoactivation; and (2) glutamate was released through 4, 4′-Diisothiocyanostilbene-2, 2′-disulfonic acid (DIDS)-sensitive anion channels (Sasaki et al., 2012). However, the trigger of glutamate release after ChR2 activation remained unclear.

Beppu et al. (2014) focused on the fact that ChR2 shows greater permeability for H^+^ compared to Na^+^ (Nagel et al., 2003). They suspected that glutamate was released secondary to intracellular acidification induced by H^+^ influx into Bergmann glial cells through ChR2 (Beppu et al., 2014). As expected, they demonstrated that ChR2 opening induced the intracellular acidification and triggered the release of glutamate. Furthermore, Beppu et al. (2014) found the intracellular acidification of Bergmann glial cells during the cerebral ischemia. They hypothesized that the intracellular acidification of Bergmann glial cells triggered glutamate release, which exacerbated neuronal damage by ischemia. They carried out a series of experiments in an attempt to counteract this pathologic condition. They showed that the illumination on ArchT-expressing Bergmann glial cells induced H^+^ efflux. Furthermore, the illumination on these cells mitigated intracellular acidification of Bergmann glial cells during the ischemia and suppressed glutamate release. Subsequently, neuronal damage was reduced.

These experiments suggested that ChR2 and ArchT could be used as tools to manipulate intracellular pH, and that intracellular acidification of astrocytes induced glutamate release.

## Analysis of Glial Cell Intracellular Calcium Level

In studies of glial cells, especially astrocytes, the fluctuation of the intracellular calcium level has been analyzed to monitor the cellular response to the external stimulus (Wang et al., 2006; Schummers et al., 2008). Studies have been focused on the temporal relationships between intracellular calcium responses and other physiological or pathological events, such as changes in blood flow (Takano et al., 2006) or synaptic activity (Panatier et al., 2011; Min and Nevian, 2012), and if calcium transients occur before or after such events. It was found that the timing of calcium transient in the soma of astrocytes differed from that in their fine processes (Shigetomi et al., 2010; Otsu et al., 2015). These observations enhanced opportunities for *in vivo* research of calcium transients in astrocyte fine processes, and for re-investigation of the temporal relationships between those changes and other events (Volterra et al., 2014).

An essential requirement to visualize calcium transients in astrocytic fine processes is the expression of sufficient amounts of calcium indicators. To satisfy this requirement, Yellow Cameleon nano50, a ratiometric calcium indicator, was expressed sufficiently and specifically in astrocytes using the KENGE-tet system. Calcium indicator amount was high enough to monitor astrocytic calcium dynamics *in vivo* using two-photon microscopy (Kanemaru et al., 2014). The results demonstrated calcium transients in resting state astrocytes under anesthesia and that most (80%) transients appeared in the fine processes. The half-life of calcium transients was about 40 s. In response to sensory stimulation, intracellular calcium level increased in both processes and soma. Meticulous observation revealed that the calcium transient initially occurred at the distal end of fine processes and spread toward the soma. This suggested that astrocytes detected extracellular environmental changes, such as neural activity, via fine processes.

## Conclusion

The continuous improvement of observation and manipulation probes in glial cell research is of great importance. One of the important points is the expression of sufficient amounts of these probes specifically in glial cells. The KENGE-tet system is a useful tool for this purpose.

## Conflict of Interest Statement

The authors declare that the research was conducted in the absence of any commercial or financial relationships that could be construed as a potential conflict of interest.

## Footnotes

^1^http://mus.brc.riken.jp/en/

^2^http://jaxmice.jax.org/list/ra2600.html
